# Percutaneous Injection of Fluids into the Trachea, Extension of Them into the Lung, and Effect of Them on the Lung and the Whole Organism

**Published:** 1887-07

**Authors:** 


Percutaneous Injection of Fluids into the Trachea, Extension of them into the Lung, and Effect on the Lung and the Whole Organism.— Dr. Sehrwald, of Jena, in the *Deutsches Archiv. für Klin. Med., Bd.* XXXIX., p. 163, reports a series of experiments, and summarizes the results as follows:

1. The perforation of the trachea of a dog with a Pravaz syringe is not dangerous, is easy and not painful.

2. The re-action of the entry of fluids is cough. This can be diminished by warming the fluids, by narcosis, and by custom.

3. The volume of the introduced fluid may be for a dog 750 gr.

4. Efficient fluids are sublimat., 1 : 5,000; acid bor., 5 : 100; acid salicyl., 1 : 100.

5. By varying the position, the fluid can be introduced into every part of the lung.

6. The fluid pervades the alveoli, the peribronchial region, the bronchial glands and the kidneys.

7. The absorption by the lung is quicker than that of the tractus intestinalis, or of the subcutaneous tissues.

8. The lung can absorb in five days four times its own weight.

9. The effect of drugs is, therefore, quicker if they are introduced by the lungs than by any other way.

10. Injecting into the lungs is like injecting directly into the circulation.

The author hopes that it will be possible to use this method for the treatment of lung diseases.—*Journal of Laryngology and Rhinology.*

